# The other possible mechanism of the benefits of RAAS inhibition in the athogenesis of COVID‐19

**DOI:** 10.1002/clc.23494

**Published:** 2020-11-11

**Authors:** Neelesh Gupta, Krunalkumar Patel, Rajeev Gupta

**Affiliations:** ^1^ Department of Internal Medicine Nazareth Hospital Philadelphia Pennsylvania USA; ^2^ Department of Internal Medicine St Mary Medical Center Langhorne Pennsylvania USA; ^3^ Mediclinic Al‐Jowhara Hospital Al Ain UAE

Letter to the Editor,

We read with interest the meta‐analysis of association of angiotensin‐converting enzyme inhibitors (ACEIs)/angiotensin II receptor blockers (ARBs) with the risk and severity of COVID‐19.^1^ Another possible explanation of the beneficial effect of renin‐ angiotensin‐aldosterone system (RAAS) inhibition on the severity of COVID‐19 is via increasing tissue kallikrein and upregulating ACE‐2 receptors.

It is proven that tissue kallikrein prevents apoptosis and ventricular remodeling after myocardial infarction.^2^ Since organ injury determines mortality in hypertensive patients infected with SARS‐CoV‐2, increased bradykinin levels induced by ACE inhibition may provide more protection than harm in cardiovascular disease (CVD)‐comorbid COVID‐19 patients.

Animal models of infection with SARS‐CoV showed that ACE‐2 downregulation resulted in pro‐inflammatory responses, including lung injury and impairment of cardiac contractility.^3^ If SARS‐CoV‐2 infections progress like SARS‐CoV, the virus may deteriorate a patient's condition by lowering ACE‐2 expression and by decreasing the degradation of angiotensin II (Ang‐II). Overabundance of Ang‐II has multiple deleterious cardiovascular consequences including elevating blood pressure, cardiomyocyte apoptosis, cardiac infiltration by macrophages, and secretion of pro‐inflammatory cytokines. Secondly, less ACE‐2 reduces the formation of angiotensin (1‐7) (Ang 1‐7) and its CVD‐protective vasodilatory, anti‐inflammatory, antifibrillatory, and antiproliferatory effects.^4^ In particular, Ang 1‐7 can diminish Interleukin‐6 (IL‐6), Tumor necrosis factor‐alpha (TNF‐α), macrophage infiltration, vascular cell adhesion protein.

Ferrario and co‐workers have showed that ACEI caused a 1.8‐fold rise of plasma Ang 1‐7 concentrations and an approximately 25% increase of ACE‐2 expression in the left ventricle.^5^ Thus, the usage of ACE inhibitors and ARBs may have a potential benefit in preventing COVID‐19‐triggered organ damage via its upregulation of Ang 1‐7, depletion of Ang‐II, and upregulating ACE‐2 receptors.

## CONFLICT OF INTEREST

The authors declare no potential conflict of interest.
